# Influence of Psychological Factors in Federated Futsal and Lifeguard Athletes, Differences by Gender and Category

**DOI:** 10.3389/fpsyg.2021.680419

**Published:** 2021-06-21

**Authors:** Francisco Cano-Noguera, Ricardo José Ibáñez-Pérez, Francisco Cavas-García, Alfonso Martínez-Moreno

**Affiliations:** INGESPORTFI Research Group, Department of Physical Activity and Sports, University of Murcia, Murcia, Spain

**Keywords:** optimism, competitive anxiety, engagement, resilience, sport, federated

## Abstract

This research aims to analyse the differences in optimism, resilience, engagement and competitive anxiety as a function of the sport modality practiced in lifeguarding (individual sport) and futsal (team sport); the sport category by age (cadet or youth) and gender. The LOT-R optimism questionnaire, the Connor-Davidson Resilience Scale (CD-RISC-10), the Utrecht Work Engagement Scale (UWES) and the Competitive Anxiety Scale (SAS-2) were applied to a sample of 189 participants (139 men and 50 womwn) aged between 14 and 17 years. The following statistical tests are performed: Cronbach's alpha, Pearson's linear correlation, Student's t-test, Kolmogorov-Smirnov test, Levene's test and multivariate linear regression. The data indicate that there are significant gender differences in total anxiety (p <0.001) and all its dimensions (somatic, worry, worry-free), also in optimism and pessimism (*p* < 0.001), as well as in total engagement (*p* = 0.051) and the absorption dimension (*p* < 0.001). When comparing the sample by sport categories, there are statistically significant differences in somatic anxiety (*p* = 0.036) and deconcentration (*p* = 0.034), as well as in LOT-total (*p* ≤ 0.001) and pessimism (*p* ≤ 0.001). In relation to the sport modalities, lifeguards show more anxiety 38.39 (0.49) and more commitment 4.58 (0.87) while futsal athletes reach higher scores in deconcentration 8.45 (2.29). It is concluded that the variables of commitment and resilience had a statistically significant positive effect, and the category of <16 years had a statistically significant negative effect, so the lower the category, the higher the optimism.

## Introduction

Sport research related to stress and well-being factors has been widely developed in recent years (López, 2015; Barbosa and Urrea, 2018), considered as important in different sports (Moreno-Murcia et al., 2006; Cano et al., 2019). Previous studies have highlighted the importance of optimism as a facilitating aspect of resilience (Ferrando et al., 2002; Aranzana et al., 2016; Martínez-Moreno et al., 2020), and competitive stress as a possible inducer of specific development in sport training stages, while also predicting how combinations of other variables could influence the athlete. It is the resilience and optimism of the individual coping strategies, which the athlete uses, that most help him or her (Galli and Vealey, 2008; Fletcher and Sarkar, 2012; García et al., 2014). Some research (Almagro et al., 2011) has indicated the importance of the perceived climate, in training or with peers, of motivation to improve results, as well as the importance of resilience in sport (García et al., 2014). However, they jointly neglect, from a positive point of view, feelings of resilience, optimism and engagement, as well as from a negative point of view, competitive anxiety and perceived stress in sport.

The present study contributes to the literature on psychological elements affecting athletes by examining the competitive context in the disciplines of lifeguarding, individual sport and futsal team sport.

First, the constructs, anxiety, resilience, optimism and engagement are analyzed in the sport domain.

### Anxiety, Types and Effects on Sportsmen and Sportswomen

Athletes during competitions are exposed to an environment conducive to generate anxiety symptoms. Anxiety can be defined as a future emotional state characterized by a sense of apprehension, worry and lack of control over one's emotional response (Otto et al., 2010). The term competitive anxiety was coined by Martens (1977) to refer to sport-specific anxiety. Competitive anxiety, which has been examined globally, is now considered an emergent and transient property of the athlete with respect to competition (Anshel, 1995), with two levels of response, which are cognitive and somatic (Ramis et al., 2010). In this direction, Weinberg and Gould (2010) differentiate on the one hand trait anxiety is seen as an acquired behavioral tendency or inclination and is part of the personality or character of athletes. On the other hand, state anxiety is seen as a constantly changing emotional component. These different dimensions of competitive state anxiety may be independently and differentially related to performance (Liştea et al., 2017). Anxiety is considered an endogenous determinant of attention (Castillo, 2009). Therefore, attention should be considered essential as competitive situations will always generate a degree of anxiety in athletes that may affect their performance due to the impairment of the attentional process (Moran, 2012). The study of competitive anxiety is very relevant given its impact -especially when negative- on athletes' performance and has a long history in sport psychology from its beginnings (Smith et al., 1990) to more recent research (Ponseti et al., 2016; González et al., 2017; Castro-Sánchez and Zurita-Ortega, 2019; Castro-Sánchez et al., 2020; Jaramillo et al., 2020; Núñez et al., 2020). Anxiety responses are accompanied by increased physiological arousal, mediated by the autonomic nervous system (Cashmore, 2008). Anxiety in sport has been extensively analyzed in different studies (Hamidi and Besharat, 2010; Correia and Rosado, 2018), as well as in sport performance being of great interest to researchers and coaches (Liştea et al., 2017).

### Resilience in Sport

Resilience has become increasingly important in recent years in the field of sport (Zurita-Ortega et al., 2018). According to the theory of Fletcher and Sarkar (2012), based on psychological resilience in Olympic champions, it would be the positive evaluation of the stressful situation shaped by a positive personality, motivation, confidence, concentration and social support, which provide effective decisions and reflections aimed at commitment to the task and increased effort. Therefore, a high level of resilience allows for greater and better adaptation to the euphoria of victory or the disappointment of defeat, using more adaptive coping strategies (García et al., 2014). Being resilient can improve sport performance and vice versa (Chacón-Cuberos et al., 2016). Providing resilience and perseverance leads to greater emotional calm and better planning (Laborde et al., 2017). The capacity to be resilient is twofold, one of which is resilience in the face of conflict, and the other is a positive behavior or attitude toward conflict (Vanistendael and Lecomte, 2002). Together with the optimistic person who has a more adaptive approach to reality than the pessimist (Flórez-Lozano, 2006), we can feel the contribution that optimism makes to resilience (Scheier and Carver, 2003), feeding into it to achieve greater resilience.

### Optimism and Sport

Based on the trait theory formulated by Scheier and Carver (1985), optimists are people who have positive expectations and perceptions about their life, while pessimists tend to represent their life negatively and the future as undesirable. Based on Scheier and Carver (2003) guidelines on dispositional optimism, having positive expectations in the face of difficulties increases efforts, and vice versa. Optimism, as a personality construct, is present in athletes and women in pressure situations (Seligman, 2004). When the athlete or woman shows more optimism, she or he presents better sports results and a higher performance (García-Naveira and Díaz, 2010; Londoño et al., 2011). Several studies have pointed out the relationship between resilience and optimism (Yu and Zhang, 2007; Parkes and Mallet, 2011; Souri and Hasanirad, 2011; González-Arratia et al., 2012; Freche, 2013). For Ortín et al. (2013) adolescent handball players with an optimistic profile present less anxiety-state, as well as older players are more optimistic than younger players (García-Naveira, 2008).

### Sport Engagement

The above elements or variables, together with engagement, can help or harm athletes if they are not taken into account. Engagement would be a persistent motivational state experienced by athletes in relation to their sport practice and would involve three dimensions: vigor, dedication and absorption. The vigor dimension refers to high levels of energy, high persistence and a strong desire to put effort into training/competition. Dedication is identified with high levels of meaning associated with training/competition, as well as pride and identification with the sport they are playing. Finally, absorption implies high levels of concentration along with the feeling that time is flying by and one is carried away (Schaufeli et al., 2001). Engagement is an optimal state in sport and a type of well-being, which can influence performance as it is one of the components of mental toughness (Crust, 2007). Very little work has applied the study of engagement in the sport domain (Lonsdale et al., 2007b; Hodge et al., 2009; De Freese and Smith, 2013) and it needs to be studied to learn more about how it relates to other aspects (Lonsdale et al., 2007a).

### Rationale for the Research

The basis for this research is that some studies have focused on individual sports (Cano et al., 2019), such as swimming, or group sports (Ruiz et al., 2012) such as football. Recently two studies have compared individual and team sports (Reche et al., 2019; Reche-García et al., 2020) but not lifesaving and futsal, which are addressed in this study. It is clear from the scientific literature that individual sports athletes have different characteristics from team sports athletes, in relation to personality (Nia and Besharat, 2010; Raharjo et al., 2018), in terms of the use of mental skills (Kajbafnezhad et al., 2011) in mental toughness (Kumar, 2017) in relation to manifesting anxiety and depression are athletes in individual sports are more likely to present them than those in team sports (Pluhar et al., 2019), in addition in team sports passion profiles are higher than those of individual sports (Kovacsik et al., 2020).

Lifeguarding is based on individual performances with which the participants achieve an individual result and at the same time award points in a ranking to their team, but unlike futsal, there is no collaboration, no joint strategy, no zone defense or collective attack, not even a possibility of verbal communication that takes place in an aquatic environment. Some of the events that take place in the swimming pool are: 200 m hurdles, 100 m combined, 100 m fins, 4 × 50 rescue tube, 4 × 50 hurdles, etc.

Therefore, the working hypotheses will be:

H1, men have less anxiety, commitment and resilience than girls.

H2, youth athletes are more optimistic than cadet athletes.

H3, futsal athletes (collective sport) show lower levels of commitment and resilience than lifeguards (individual sport).

H4, futsal players have less anxiety than lifeguards.

All of the above leads us to the objective of the study, which focuses on finding out the existing differences between lifesaving (individual sport) and futsal (team sport) from a psychological perspective, as well as determining differences in relation to the sporting categories, sex and age of the participants, providing answers to the different hypotheses put forward.

## Materials and Methods

### Design and Participants

A cross-sectional, observational and quantitative design was carried out, selecting the sample in a non-probabilistic or intentional way. The study, Table 1, consisted of a total of 189 young lifeguards and futsal players, corresponding to the youth categories, under 18 years of age (16–17) and cadets, under 16 years of age (14–15). Of which 103 participants were lifeguards and 86 futsal players, 50 girls and the remaining 139 men.

**Table 1:** Descriptive statistics of the sample.

	***n***	**%**
**Sex**		
Female	50	26.5
Male	139	73.5
**Sport**		
Futsal	86	45.5
Lifeguard	103	54.5
**Category**		
Youth	86	45.5
Cadets	103	54.5

### Procedure

All the athletes were asked, through their coaches, to collaborate in the research. All the minors, in the lifeguard modality, in the course of the processing of the sports license for the year included a section in which they accepted to participate as sample subjects in possible non-invasive studies that could be carried out. Once informed consent had been obtained from the parents/guardians/legal guardians of the futsal players, all those who wished to participate in the study filled in the questionnaires prior to the celebration of a Spanish championship for the lifeguards and before a league match for the futsal players. Data were collected before the covid-19 pandemic, from the lifesaving athletes, on the day of the competition, in an after-breakfast meeting, all were concentrated in a hotel, and from the futsal players, before the pre-match talk, about 2 h before the lifesaving competition and matches. Their preparation and level of physical work was part of the peak performance phase, taking into account that all participants trained 3–4 h per week. The study was conducted under the guidelines of the Declaration of Helsinki, the design, observational, did not contain any ethical aspects that required prior authorization from the Bioethics Committee of the University of Murcia, Spain.

### Instruments

The questionnaire consisted of a total of 54 items comprised of four separate and established questionnaires measuring resilience, competitive anxiety, optimism and engagement, as well as socio-demographic data. Resilience (RS) was measured using the Connor-Davidson Resilience Scale (CD-RISC-10) (Serrano-Parra et al., 2012). The scale has an internal consistency Cronbach's alpha of 0.89 and with a single dimension consisted of 10 statements (e.g., “I know how to adapt to change”) with participants responding on a five-point Likert scale, ranging from 0 (“Strongly disagree”) to 4 (“Strongly agree”).

The Competitive Anxiety Scale (SAS-2) (Ramis et al., 2010) was used to measure anxiety, with an internal consistency using Cronbach's alpha coefficient of 0.83 for the somatic (AS) subscale, 0.78 for the worry subscale (A-C) and 0.73 for the deconcentration (A-D) subscale. It consists of 15 statements preceded by the phrase “Before the competition.” (e.g., “. I find it difficult to concentrate on the match or competition”). Responses were again given on a four-point Likert scale, ranging from 1 (“Nothing”) to 4 (“Much”). Somatic anxiety is understood as anxiety that is perceived by the athlete for no apparent reason, worry anxiety as anxiety that can be justified by a specific situation, deconcentration anxiety as anxiety that due to its importance reduces the involvement in the task at hand and total anxiety as the final result of linking all of them together.

Optimism was assessed with the Life Orientation Test-Revised (LOT-R) (Scheier et al., 1994) in its Spanish version (Ferrando et al., 2002), to measure dispositional optimism or generalized predisposition toward expectations of positive outcomes. The LOT-R has good psychometric properties, Cronbach's reliability of 0.70 and 0.69 for optimism and pessimism respectively. The LOT-R questionnaire consists of 10 items (e.g., “In difficult times, I usually hope for the best”), of which four are control items and of the remaining six, three are written in a positive direction (optimistic direction) and three in a negative direction (pessimistic direction) with responses given on a five-point Likert scale, ranging from 0 (“Strongly disagree”) to 4 (“Strongly agree”). Likewise, LOT-R Optimism (LOT-O) is understood as the ability of the athlete or woman to have a positive view of situations, LOT-R Pessimism (LOT-P) as the ability to see the negative side of situations and LOT-R Total (LOT-T) as a combination of both. For the sample analyzed, optimism obtained a Cronbach's alpha of 0.79 and for pessimism 0.84.

The engagement questionnaire was the Utrecht Work Engagement Scale (UWES) (Guillén and Martínez-Alvarado, 2014), with a Cronbach's alpha reliability of 0.93 and consisting of 9 items. This questionnaire measures the three dimensions of engagement, which are vigor (UWES-V) (e.g., “I feel energized at work”), dedication (UWES-D) (e.g., “My work inspires me”) and absorption (UWES-A) (e.g., “I feel disengaged when I am working”). Each item is answered on a Likert-type scale, ranging from 0 (“Never”) to 6 (“Always”).

### Statistical Analysis

For the descriptive statistical analysis of the sample, the number of cases present in each category and the corresponding percentages of the qualitative variables were obtained. For the quantitative variables, the following descriptive statistics were used: minimum, maximum, mean and standard deviation (SD). Cronbach's alpha was calculated in order to check the reliability of the different scales within this particular sample, and correlations between variables were calculated using Pearson's linear correlation coefficient (r). In addition, for quantitative variables, the t-Student test was performed for the comparisons of means between two groups. Assumptions of normality and uniformity of the variances required for mean comparisons were tested using the Kolmogorov-Smirnov test and the Levene test, respectively. Finally, a multivariate linear regression model was developed to determine the possible effects of sex (male or female), sport modality (lifeguards or futsal) and category (cadet or youth) on the resilience, anxiety and engagement scales. Statistical analyses were performed using SPSS 25.0 for Windows (IBM, New York, NY, USA). Statistical significance was defined as *p* < 0.05.

## Results

The mean (typical deviations), Cronbach's alpha reliability indexes, and the correlations between the different subscales of the questionnaires used that address each variable chosen. Internal consistency rates were all higher than 0.79, indicating high reliability.

In Table 2, we can see that A-T correlated statistically significantly and positively with A-S, A-C, total engagement (UWES-T), UWES-D and UWES-A dimensions. But negatively and statistically significantly with LOT-O and LOT-P. A-S correlated positively and statistically significantly with A-C, UWES-T, UWES-D and UWES-A and significantly but negatively with A-D, LOT-O and LOT-P. A-C had a positive and statistically significant relationship with UWES-T, UWES-D and UWES-A and was negatively correlated with A-D, LOT-O and LOT-P. The A-D dimension showed a positive and significant correlation with the LOT-O and LOT-P and had a negative correlation with LOT-T, UWES-T, UWES-D, UWES-A and RS.

**Table 2:** Means, standard deviations, reliability and Pearson's correlation of the scales.

	**Mean (SD)**	**Cronbach's Alpha**	**A-T**	**A-S**	**A-C**	**A-D**	**LOT-T**	**LOT-O**	**LOT-P**	**UWES-T**	**UWES-V**	**UWES-D**	**UWES-A**
A-T	34.8 (5.66)	0.855	1										
A-S	10.99 (3.1)	0.801	0.892^**^	1									
A-C	16.66 (3.6)	0.794	0.852^**^	0.762^**^	1								
A-D	7.15 (2.36)	0.866	−0.076	−0.338^**^	−00.483^**^	1							
LOT-T	15.47 (2.8)	0.835	−0.021	0.092	00.102	−0.327^**^	1						
LOT-O	6.7 (2.31)	0.791	−0.474^**^	−0.558^**^	−0.506^**^	0.370^**^	0.484^**^	1					
LOT-P	3.23 (2.63)	0.845	−0.394^**^	−0.588^**^	−0.553^**^	0.672^**^	−0.639^**^	0.364^**^	1				
UWES-T	4.39 (0.98)	0.811	0.199^**^	0.266^**^	0.235^**^	−0.232^**^	0.300^**^	−0.033	−0.348^**^	1			
UWES -V	4.05 (1.21)	0.859	−0.027	−00.019	0.005	−0.048	0.188^**^	0.12	−0.095	0.856^**^	1		
UWES -D	4.48 (1.03)	0.881	0.157^*^	00.234^**^	00.205^**^	−0.243^**^	0.317^**^	−0.014	−0.350^**^	0.935^**^	0.751^**^	1	
UWES -A	4.63 (1.14)	0.832	0.401^**^	00.497^**^	0.417^**^	−0.328^**^	0.289^**^	−0.200^**^	−0.483^**^	0.831^**^	0.472^**^	0.716^**^	1
Resilience	27.51 (4.26)	0.794	−0.121	00.01	−0.026	−0.264^**^	0.460^**^	0.254^**^	−0.266^**^	0.170^*^	0.265^**^	00.162^*^	.011

*A-T, Anxiety Total; A-S, Anxiety Somatic; A-C, Anxiety Concentration; A-D, Anxiety Deconcentration; LOT-T, Lot Total; LOT-O, Lot Optimism; LOT-P, Lot Pessimism; UWES-T, UWES Total; UWES-V, UWES Vigor; UWES-D, UWES Dedication; UWES-A, UWES Absorption*.

* *p < 0.05,*

** *p < 0.01*.

As for the LOT-R scale, the LOT-T correlated positively and statistically significantly with the LOT-O, UWES-T, UWES-V, UWES-D, UWES-A and RS, however, it correlated negatively and statistically significantly with the LOT-P. The LOT-O dimension was positively and statistically significantly correlated with LOT-P and SR and negatively and statistically significantly correlated with UWES-A. The LOT-P dimension had a negative and statistically significant relationship with the UWES-T, UWES-D, UWES-A and resilience.

The UWES-T showed positive and statistically significant differences with the UWES-V, UWES-D, UWES-A and SR. The UWES-V dimension showed positive and statistically significant differences with UWES-D, UWES-A and RS, and the UWES-D dimension showed positive and statistically significant differences with UWES-A and RS.

Focusing on the differences between males and females in the sample, Table 3 presents the results of the SAS-2, LOT-R, UWES and RS scales based on gender. There were statistically significant and positive differences in terms of A-T, A-S and A-C, with males reaching higher values, while there were statistically significant and negative differences in favor of females in terms of A-D. LOT-O and LOT-P also showed positive and statistically significant differences in favor of girls. Finally, there were also statistically significant differences in relation to UWES-T, where boys scored higher, as well as in relation to UWES-A, where girls scored higher.

**Table 3:** Correlations of anxiety, LOT, UWES and resilience in relation to sex.

	**Sex**		**Mean difference**	**Test** ***t*****-student**		***d***
	**Men (*n* = 139)**	**Women (*n* = 50)**		***t*_**(379)**_**	***p-*value**	
**Anxiety**						
Total	38.36 (0.48)	33.53 (6.11)	4.83	5.581	** <0.001**	0.63
Somatic	13.36 (0.48)	10.14 (3.21)	3.22	7.051	** <0.001**	0.80
Concern	18.96 (1.80)	15.83 (3.73)	3.13	5.702	** <0.001**	0.64
Deconcentration	6.04 (1.80)	7.55 (2.41)	−1.51	−4.047	** <0.001**	−0.46
**LOT**						
Total	15.96 (0.73)	15.29 (3.22)	0.67	1.461	0.146	0.17
Optimism	5.44 (1.15)	7.15 (2.46)	−1.71	−4.74	** <0.001**	−0.54
Pessimism	1.48 (1.31)	3.86 (2.70)	−2.38	−5.984	** <0.001**	−0.68
**UWES**						
Total	4.62 (0.86)	4.31 (1.01)	0.32	1.965	**0.051**	0.22
Vigor	3.96 (1.21)	4.08 (1.22)	−0.12	−0.618	0.537	−0.07
Dedication	4.71 (0.82)	4.40 (1.09)	0.30	1.8	0.073	0.20
Absorption	5.20 (0.82)	4.43 (1.17)	0.77	4.271	** <0.001**	0.48
**Resilience**	27.72 (2.96)	27.43 (4.64)	0.29	0.41	0.682	0.05

*p < 0.001. The bold values indicates statistically significant differences*.

Regarding the sport category variable, higher A-S was found in the juvenile group (<18 years) and higher A-D in the cadets (<16 years), as shown in Table 4. The juvenile group scored higher on LOT-T, while the cadets scored higher on LOT-P. There were no differences in any of the engagement dimensions, but the juvenile group had a higher RS ability than the cadets.

**Table 4:** Correlations of anxiety, LOT, UWES and resilience in relation to category.

	**Category**		**Average difference**	**Test** ***t*****-student**		***d***
	**Youth (*n* = 86)**	**Cadets (*n* = 103)**		***t*_**(379)**_**	***p*-value**	
**Anxiety**						
Total	35.36 (5.07)	34.34 (6.09)	1.02	1.237	0.218	0.14
Somatic	11.51 (2.92)	10.56 (3.20)	0.95	2.111	**0.036**	0.24
Concern	17.09 (3.47)	16.29 (3.68)	0.80	1.53	0.128	0.17
Deconcentration	6.76 (2.05)	7.49 (2).55)	−0.73	−2.137	**0.034**	−0.24
**LOT**						
Total	16.36 (2.20)	14.72 (3.03)	1.64	4.189	** <0.001**	0.47
Optimism	6.79 (2.45)	6.62 (2.20)	0.17	0.501	0.617	0.06
Pessimism	2.43 (2.09)	3.90 (2.85)	−1.47	−3.983	** <0.001**	−0.45
**UWES**						
Total	4.46 (0.89)	4.33 (1.05)	0.13	0.913	0.362	0.10
Vigor	4.04 (1.17)	4.06 (1.25)	−0.02	−0.088	0.93	−0.01
Dedication	4.55 (0.92)	4.43 (1.11)	0.12	0.771	0.442	0.09
Absorption	4.79 (1.01)	4.50 (1.23)	0.29	1.769	0.078	0.20
**Resilience**	28.31 (3.89)	26.84 (4.45)	1.48	2.408	**0.017**	0.27

*p < 0.001. The bold values indicates statistically significant differences*.

When the sample was divided according to sport modality (lifeguard/futsal), there were statistically significant differences between the groups in A-T, and its dimensions, A-S, A-C and A-D, Table 5. A-T, A-S and A-C were higher in the lifeguard group, while A-D was higher in the futsal group. There were statistically significant differences in LOOT-T, LOT-O and LOT-P, with the lifeguard group scoring higher in terms of LOT-T, but in LOT-O and the LOT-P, the highest scores were found in the futsal group. In the UWES-T scale, as well as in its dimensions UWES-D and UWES-A, statistically significant differences were found, where the lifeguard group had higher scores than the futsal group in these three dimensions. There were no significant differences in the remaining dimensions of the scales.

**Table 5:** Correlation anxiety, LOT, UWES, and resilience depending on the sport modality.

	**Sport**		**Average difference**	**Test** ***t*****-student**		***d***
	**Futsal (*n* = 86)**	**Lifeguard (*n* = 103)**		***t*_**(379)**_**	***p-*value**	
**Anxiety**						
Total	30.51 (6.03)	38.39 (0.49)	−7.88	−13.219	** <0.001**	−1.49
Somatic	8.13 (2.40)	13.39 (0.49)	−5.26	−21.721	** <0.001**	−2.46
Concern	13.93 (3.31)	18.93 (1.81)	−5.00	−13.17	** <0.001**	−1.49
Deconcentration	8.45 (2.29)	6.07 (1.81)	2.39	7.997	**<** **0.001**	0.90
**LOT**						
Total	14.97 (4.03)	15.88 (0.72)	−0.92	−2.271	**0.024**	−0.26
Optimism	8.27 (2.37)	5.39 (1.17)	2.88	10.866	** <0.001**	1.23
Pessimism	5.30 (2.30)	1.50 (1.31)	3.80	14.233	** <0.001**	1.61
**UWES**						
Total	4.17 (1.07)	4.58 (0.87)	0.41	−2.89	**0.004**	−0.33
Vigor	4.18 (1.24)	3.95 (1.19)	0.23	1.319	0.189	0.15
Dedication	4.27 (1.20)	4.66 (0.83)	−0.39	−2.627	**0.009**	−0.30
Absorption	4.05 (1.19)	5.12 (0.83)	−1.07	−7.217	** <0.001**	−0.82
**Resilience**	27.00 (5.49)	27.93 (2.81)	−0.93	−1.503	0.134	−0.17

*p < 0.001. The bold values indicates statistically significant differences*.

To determine in LOT-R the effects of the variables sex, type of sport (lifeguard-futsal) and sport category (<18 and <16 years) and anxiety, UWES and RS, a linear regression was performed. The results are shown in Table 6, the model was statistically significant *F*_(6;182)_ = 14.1; *p* < 0.001, explaining 31.7% of the explanatory variance. The variables of commitment and resilience had a significant positive effect, and the category of <16 years had a significant negative effect, so the lower the category, the higher the optimism.

**Table 6:** Linear regression.

	***B* (ET)**	**Beta**	***t***	***p*-value**
Sex (Male, Female)	−0.37 (0.47)	−0.059	−0.8	0.425
Sport (Lifeguard, Futsal)	0.53 (0.56)	0.095	0.958	0.339
Category (Cadets, Youth)	−1.17 (0.36)	−0.209	−3.276	**0.001**
UWES	0.63 (0.18)	0.222	3.48	**0.001**
Anxiety	−0.06 (0.04)	−0.129	−1.441	0.151
Resilience	0.24 (0.04)	0.358	5.433	** <0.001**

*F_(6, 182)_ = 14.1; p <0.001; R2 = 0.317. The bold values indicates statistically significant differences*.

## Discussion

In sport competition, it is essential to know and control the factors (physical, technical and psychological) that influence performance (Morillo et al., 2016). Going deeper into what Cerin (2003) indicates that the pre-competitive state of the athlete must be analyzed from an interconductual approach to human behavior, which includes other related emotions in addition to anxiety, taking the field model as a reference, looking for relationships with other variables.

The aim of the study focuses on finding out the existing differences between the lifesaving (individual sport) and futsal (team sport) modalities; from a psychological perspective, as well as determining differences in relation to the sport categories, sex and age of the participants, responding to the different hypotheses put forward.

On analyzing the correlations of the different scales used in the study, we found a relationship between the level of LOT-T and SR, as well as between the UWES-T and SR. Likewise, we found a negative correlation between the level of A-D and SR, data that support previous studies Trigueros et al. (2020) and Reche et al. (2018). In addition, the study has provided other interesting data such as a correlation between LOT-O level and UWES-D level with SR, supporting previous studies such as Vallarino and Reche-García (2016) with hockey players and Ruiz et al. (2012) with football players; Trigueros et al. (2020) with volleyball players and Reche et al. (2018) with fencers. Similarly, we found a negative correlation between the level of A-D and RS, which was again consistent with the findings of previous studies Reche et al. (2019).

The present data reflected a higher level of A-T and UWES-T in boys, as well as lower levels of A-D and higher levels of LOT-O in girls. Thus, partly corroborating H1, boys have lower A-T, UWES-T and RS than girls. This finding is in line with some previous studies (Martens et al., 1990; Clifton and Gill, 1994) and at the same time does not agree with Reche et al. (2018) and Aranzana et al. (2016) who found no differences between sexes. As for the second part of the hypothesis, it is not corroborated, as girls have higher LOT-O scores, a fact explained by Stach (2006) in sociobiological terms: women, as the mother of the species, must find support mainly in themselves and in their own activities. These data are in opposition to other studies, albeit conducted in the academic field, where Extremera et al. (2007) found that male students showed a stronger optimistic tendency than female students. However, some studies have found that there were no gender differences, for example: Tan and Tan (2014), Aranzana et al. (2016), Hinz et al. (2017), Reche et al. (2018), and Cnen et al. (2020). Furthermore, other authors indicate differences in the interpretation of symptoms in relation to the type of sport and competition experience (Mellalieu et al., 2004). Thus, this variability of data could be marked by the cross-sectional nature and sport modalities of the study, and further longitudinal studies are needed to clarify the nature of these differences.

When analyzing the data according to sport categories: youth (<18 years) and cadets (<16 years), the data show a similar result to previous studies, such as those of Vallarino and Reche-García (2016), who found a higher level of optimism in the under-18 age groups compared to the under-16s. However, other studies, such as Bohórquez and Checa (2017), Sánchez et al. (2012), and Reche et al. (2018), have shown different patterns to the present research, which found higher levels of somatic anxiety and resilience in young people and greater deconcentration and pessimism in under-16s. Kristjánsdóttir et al. (2019) found no significant differences in futsal players related to competitive anxiety or resilience. These results coincide with those presented by Reche-García et al. (2020) where they do not find differences related to the age variables of the participants. In relation to H2, youth category athletes are more optimistic than cadet category athletes, H4 is corroborated. Endorsing previous studies, Vallarino and Reche-García (2016), who found a higher level of LOT-T in groups <18 years compared to <16 years. This difference may be influenced by the fact that cadet athletes, because of their age, have an enthusiastic commitment that represents an individual's feelings of attraction and dedication toward an entity (in this case sport modality) and youth athletes may have a limited commitment that reflects an individual's feelings of obligation and passive responsibility toward an entity (or sport team) (Scanlan et al., 2016). However, other studies, such as Bohórquez and Checa (2017), Sánchez et al. (2012), and Reche et al. (2018), have shown different patterns than the present research, finding higher levels of A-S and RS in younger athletes and higher A-D and LOT-P in <16 years old. Kristjánsdóttir et al. (2019) find no significant differences in futsal players in relation to competitive A-T or RS. These discrepancies between the different studies could be conditioned by the competitive level of the athletes (local-autonomous-national-international), as these levels of competition can enhance and/or generate different responses in the variables analyzed.

When correlating the different scales in terms of sport modality, in A-T, LOT-T, UWES-T and RS, it is the lifeguard athletes who show higher scores than the futsal athletes. Which determines that H3, futsal athletes (collective sport) show a lower level of commitment and resilience than lifeguards (individual sport), H1 is confirmed. Regarding the sport modality and the relationship between collective sport (futsal) and individual sport (lifeguard), we found similar results to previous studies, such as Bohórquez and Checa (2017) and Reche et al. (2018), regarding UWES-T, A-T and LOT-T in individual sports. Likewise, the results of this study reflected a higher level of A-D and LOT-P in futsal participants (collective sport). This is in contrast to previous studies such as those of Sánchez et al. (2012) and Chacón-Cuberos et al. (2016), which found no differences in these variables according to sport modality. This is supported by the main psychological distinction between individual and team sports, which is based on the concept of responsibility (Mroczkowska, 1997), supported by the fact that personal responsibility for the result (positive or negative) is lower in team sports compared to individual sports (Laborde et al., 2016).

We believe that the results presented are due to the specific characteristics of the sports studied. On the one hand, lifeguarding is a sport in which the work is carried out individually but within a team discipline, which we believe reduces the possibility of differences between its practitioners. Whereas, in Indoor Football, the possibility of distributing responsibility among the team, we understand, is the reason for these differences between modalities. As determined by Vallarino and Reche-García (2016) who highlighted the importance of optimism and resilience in hockey players or the study by Ruiz et al. (2012) on football players.

In relation to H4, Futsal players have lower A-T than lifeguards, H3 is endorsed. Since the lifeguard athletes achieved higher scores than the futsal players. Possibly the cause could be as a consequence of the sport modality practiced (individual) as when one realizes that one is unable to achieve a goal it is probably very stressful, as stress occurs when goals become unattainable (Lazarus, 1999). Considering that lifeguarding is performed individually, but, within a team discipline, the athlete is solely responsible for the outcome. Whereas, in futsal, there is the possibility of distributing the responsibility among the different players of the team, we understand that this could be the reason for these differences between the reviewed sports modalities.

The results of the linear regression revealed the importance of the category, the level of UWES-T and the level of SR of the participants. Unlike previous studies, Sánchez-Oliva et al. (2010) or Chacón-Cuberos et al. (2016) that did not emphasize these variables and similar to the studies of Bohórquez and Checa (2017) as well as Reche et al. (2018), in individual sports such as triathlon or team sports such as hockey, which are in line with this study.

## Conclusions

The results of this study provided very valuable information about optimism, anxiety, resilience and the level of commitment of athletes <18 years old and <16 years old, both in lifeguarding and futsal. From the data of this study, it will be possible to establish a better working environment in training and provide psychological work situations so that athletes can obtain greater positive feelings, both in individual and collective sport. Providing coaches and athletes with information on the dimensions analyzed, in order to be able firstly to detect if there are risks in athletes and secondly to prevent them from appearing, with the inclusion in training and/or training of athletes of efficient tools and methodologies in the detection, prevention and reinforcement of concepts such as LOT-T and RS that help athletes and coaches to face them with solvency. Using the present research as a reference, additional future research is needed in other sports, both individual and team, before stronger conclusions can be drawn. Larger sample sizes, longitudinal studies, including pre- and post-competition observation may provide more information on the more concrete effects of these variables on athletes. Such research may also reveal whether the results of the questionnaires are different depending on the athlete's performance in an event.

It is worth noting that higher anxiety and UWES ability was observed in the lifeguarding modality, and higher levels of deconcentration and LOT-R-P were observed in futsal participants.

The study provides insight into the athletes of these two sport modalities on which to deepen future research, generate personalized lines of work and improve performance. We are also aware of the need to continue increasing the sample for future replications, and even generate sport-specific studies and comparing different competitive levels.

### Limitations of the Study

With regard to the limitations of the study, firstly, we can point out that the data have been collected through self-reporting. This is a common practice in studies, although it may lead to a bias in the participants' response, exacerbate common variance and artificially increase correlations between variables (Spector, 2006). Secondly, the sample is composed of Spanish athletes, who have their own cultural characteristics; therefore, the results obtained cannot be extrapolated to other samples. It would be interesting to carry out cross-cultural or cross-national studies to verify whether the results of our work are similar to those of other countries.

## Data Availability Statement

The raw data supporting the conclusions of this article will be made available by the authors, without undue reservation.

## Ethics Statement

Ethical approval was not provided for this study on human participants because as it was an observational study and the information was obtained through a questionnaire, in which there was no sensitive information. The ethics committee determined that there was no need for such a report. Written informed consent to participate in this study was provided by the participants' legal guardian/next of kin.

## Author Contributions

FC-N and AM-M: conceptualization, writing—review and editing, and supervision. FC-N: methodology, writing—original draft preparation, and project administration. FC-G and RI-P: software and visualization. AM-M, RI-P, and FC-N: validation. AM-M: formal analysis. FC-N and RI-P: investigation. FC-G and AM-M: resources. RI-P: data curation. AM-M, RI-P, FC-G, and FC-N: funding acquisition. All authors have read and agreed to the published version of the manuscript.

## Conflict of Interest

The authors declare that the research was conducted in the absence of any commercial or financial relationships that could be construed as a potential conflict of interest.
